# How to Write a Pathology Research Paper—Basic Principles and Beyond—A Primer for Residents

**DOI:** 10.1111/apm.70007

**Published:** 2025-02-19

**Authors:** Glen Kristiansen

**Affiliations:** ^1^ Institute of Pathology University Hospital Bonn (UKB) Bonn Germany

**Keywords:** pathology, primer, publication, scientific writing

## Abstract

Medical writing is an art but still it is usually not in the curriculum of medical students. With the beginning of scientific activity during residency, many perceive this gap increasingly, and stay behind their own expectations in their scientific productivity. Many universities offer courses to teach scientific writing and many books and article address this void, but in real life the main work to carve a readable paper out of a pile of unsorted data remains often in the hands of the scientific supervisors. This little paper tries to address this issue with a focus on typical pathology related subjects by outlining the structure of a paper and explaining typical dos and don'ts of crafting a publishable scientific paper as a pathology resident.

## 1 Introduction

In my experience working as an academic pathologist over nearly 30 years, it is relatively simple to motivate young people to get involved in scientific projects, to generate data and to analyze it, but when it comes to the writing phase, the progress often slows down, or even comes to a halt. It turns out, writing a paper is not as straightforward as people have assumed before and without an engaged mentor that supervises this process carefully, meticulously and persistently, many paper drafts written by novices end up unfinished and remain fragments. However, this supervisory work can be so time consuming or even daunting, as so many things need explanation from scratch, that not so few more senior scientists prefer to write the manuscripts themselves. This appears more economical, but deprives the young from the opportunity of experiencing the satisfaction to accomplish the final step of a scientific project, which is to write it up and to get it published. Therefore, I would like to encourage young scientists to write their paper drafts themselves and I hope that my experiences as a scientist and mentor presented in this educational paper may help to accomplish this task easier. There are of course many books, papers and courses that explain and teach the craftmanship of scientific writing but I felt that having one written by a pathologist addressing his young residents would be a valuable addition to the field [1, 2, 3]. Also, many journals provide general guidance, how to write (see e.g., https://www.nature.com/nature‐portfolio/for‐authors/write). Some aspects may appear trivial and self‐explanatory, but in real life I found, it is often the very simple things, that get messed up.

### 1.1 What Tools Are Needed?

A word processor (e.g., Word, Papers), ideally coupled to a program that manages references (e.g., Endnote, Citavi, Bookends, etc.), is desirable. You can of course work without the latter, but it will save you a lot of tedious work, if you use one. Particularly, if you aspire a career in science, you will not want to work without a citation software. It keeps the reference list in the correct order, even if you decide to insert some new citations in between others and it will transpose the reference style easily to the style of the target journal. To do this manually is possible but nerve wrecking, as every journal has its individual style to document year of publication, volume number and page numbers. If you want to start out without an automated reference managing system, it is easiest to use an *author*‐*based style*, where the citation is referenced in the text with the first authors name (e.g., Smith et al., 2005) and the reference list is kept in alphabetical order. Even if you shift whole paragraphs back or forth, this does not affect the way you cite. Many journals demand a *number*‐*based style*, where the order of references does change with changes in the citation order in the text. This is, where things become tedious, if you want to do this manually, hence my recommendation to invest time and money into a reference software.

Most paper drafts are written in a neutral font, for example, Arial or Calibri, size 12 with 1.5 to double spacing of the lines. This will ease reading and correcting the paper later and especially on print‐outs.

### 1.2 General Aspects

Work in a structured manner and build your paper section after section. By consciously sub‐structuring and fragmenting your task, it will become easier. It is often a good idea to start with the introduction, in which you clarify the scientific background and, explain the scientific/medical need and develop the aims of this paper. Others prefer to start with the Materials and Methods section, followed by the results.

In crafting a paper, practice perfect order and take care of details in your own interest, even if you are more of a creative but chaotic character. The editors and reviewers of your paper draft (and everything that is not finally accepted and published can be considered a draft) usually do not know you, your laboratory/institute and your general attitude. They will assume that the quality of the paper mirrors the quality of the experimental work; a sloppy composition, typos, poor language, wrong assignments of Legends/Figures, wrong word count, etc. might thus be taken as a witness of poor science. *Therefore, paying attention to details is of paramount importance*. Do not leave these details to your mentor, she or he will have many other things to do but try to excel by providing a paper draft, that hardly leaves room for formal criticism. If you have a legasthenic side, make use of the autocorrection function of your word processor. If English is not your native language, you may use web‐based tools like DeepL or ChatGPT to verify that you expressed yourself correctly. Let colleagues read your work and ask for feedback. Be aware that it took you months or years to collect the data of your paper and therefore you should not allow a hasty writing style compromise the publication of it. You may also consider using the services of a professional language editing provider—as competent use of language is a success factor in publishing science [4].

The *language* you use in your writing should be clear and simple if you want to be understood. Long and convoluted, highly nested sentences as well as a sophisticated and rare vocabulary may satisfy your literary ambitions, but they are out of place in scientific communication. Reading Nabokov is delightful, but he would probably have failed as a medical‐scientific writer. Obey the KISS rule (keep it simple, stupid!) without hesitation and prefer short and clear sentences. Scientific depth is not revealed in cloudy and ambiguous language; on the contrary, only the clarity and the precision of the text will make it possible to assess the depth and the impact of your research.

Take a close look at the instructions for authors of your target journal, the demanded structure, the maximum word count and the number of references allowed. Bear these constraints in mind as you write to save you later shortening and reworking your text.

## 2 The Basic Structure of a Scientific Paper

Scientific research papers follow a universally accepted linear structure: *Introduction, Material and Methods* (*M&M*), *Results, Discussion, Acknowledgements*. This is preceded by the *Abstract* and the *Title page*. There are minimal changes to the terminology or the compilation of these points, depending on the target Journal, but basically these are the main parts that need to be filled with content. In the following, we will go through these points individually. The order in which the individual parts are written is a matter of personal preference. Some like to start with the introduction in order to clarify the framework for the initial scientific questions. However, many find that it is easier to start with a description of the materials and methods used. This is followed by a clearly structured description of the results. The last and most difficult part, where everything comes together, is the discussion.

### 2.1 The Title Page

The title page starts with the *title of the scientific work* and this does indeed deserve longer consideration. A good title summarizes the main finding(s) of the study, should be precise and catchy, yet not too long and induce curiosity to read on. We are all overwhelmed with information and have to neglect things rapidly to remain functional, hence catching the reader's attention is crucial. Very few papers are read to the end, it often ends after a non‐catchy title. Very simple descriptive titles like “PD‐L1 in Colon Cancer” may do, but are too general and may serve better as an internal *running*/*short title* of the project. Better would be to be more specific, “PD‐L1 expression in high grade Colon Cancer is associated with MMR defects” is a more precise, more informative and attractive title. Keep in mind, that a good title will help your article to be found by other researchers, the prerequisite of being cited and citations are the real recognition of scientific work and merit. The same care should be applied to the selection of *keywords*. Ideally, a systematic review on the topic of your paper should be able to pick this paper up, so even though “cancer,” “pathology,” “biomarker” would most likely be accepted as keywords by the journal, they are actually useless as they are unspecific.

The title page also gives information on the *professional affiliations* of the authors, usually the institute/university, city, and country are named. The correctness of the affiliations is important and it is wise to ask all co‐authors prior to submission to verify this. Maybe you missed, that someone has moved recently?

#### 2.1.1 Author List and Authorships

It is commonly accepted good clinical practice that authors of a paper are all people who have contributed significantly to its scientific content. The most prominent author is the *first author*, who usually has carried out significant parts of the project, writes up the paper and therefore gets most of the credit later on. It will always be her or his name that will be mentioned, when this particular paper is referenced to. However, it is not a given that someone new to a research group will readily be given the honor (and task…) of a first authorship. Naturally, this position means the most work during the writing phase. It is the first author's task to compile the data and write the first draft of the paper, harmonize it first with the principal investigator (PI) of the research group, who usually is last author of this paper, then to harmonize it with all other co‐authors, compile the resulting requests for revisions and to finally submit it to the journal of choice. The *last authorship* is the second most important position on the author list. The PI/last author has usually created and masterminded the project, organized finances and contributed by her/his network to the composition of collaborating scientists and co‐authors. He or she usually is a more senior scientist, working group leader or chair of the institute, hence it does make sense that the last author is also *corresponding author*. The corresponding author is not necessarily the person, that submits or uploads a paper (this technical work could also be done by a medical student or a secretary, but is most commonly accomplished by the first author), but the person that has to be addressed by other scientists, that have questions or comments concerning this paper. This correspondence may come up even years after the publication of the manuscript, when the PhD or MD candidate or pathology resident has long left the institute, whereas the project initiating senior author is more likely to be still active. When applying for professorships, it is expected to provide the referees with a list of publications sorted by first and last authorships, rarely also by corresponding authorships, so clearly, it deserves to be vigilant to consider these author positions in time. In collaborative projects, clarifying the tasks and the authorship positions before the start of the project can save a lot of trouble later and also motivates people to become active.

#### 2.1.2 The Abstract

The abstract can be considered a highly condensed version of the whole paper and it usually also follows a very similar structure: Introduction/Aims, Material and Methods, Results, and Conclusions. Some journals prefer non‐structured abstracts‐ just skip the headers then. The word count is mostly very limited, depending on the journal, to 150–300 words. So, one really needs to focus on the main messages of the paper and eliminate all superfluous words. Compiling an informative abstract is an art! Bear in mind, that many people have massive time constraints or are shallow readers and the only part of many papers that is really being read is the abstract and maybe the final conclusion in the discussion. As with the title of a paper, we fight for the reader's attention with an attractive, catchy abstract. Therefore, the information presented here must be absolutely correct, flawless and appealing, especially the conclusion. It is advisable to write the abstract of the paper in the end, when everything else is settled.

### 2.2 The Introduction

It introduces the reader to the scientific topic and develops the question(s) that this study aims to answer. After the introduction the reader should have a fairly good idea, what was known before, why this research has been done and which scientific or clinical need it addresses. In the majority of pathology papers, the introduction either focusses on a disease, a biomarker, or a diagnostic method.

*Disease oriented introductions* will usually start off explaining why a certain disease is particularly important. The disease is either the most common, the deadliest, the least well known/most under‐researched etc. etc. of all, hence there is a huge void that needs to be filled. The explanations then become more specific, entailing clinical or diagnostic issues, and finally the core aims of the study are formulated. In *biomarker focused introductions*, the biomarker under consideration is explained, its history, its use, known data in other entities and again, finally, the open questions are explained that this study addresses. *Method oriented introductions* are rarer, and start stressing why this method was developed, what its applications are, what its strengths and weaknesses are and what should or could be improved by this study.

Whatever appears more appropriate to use, make sure you do not bore your readers by lengthy and truistic writing and but do come to the points more quickly. The very schematic approach of explaining that “cancer X is very common in the western world (Cancer statistics, 20xx), also plays a role in our country (Ref. 2), can usually be treated well (Ref. 3–6) but has issues concerning whatever (Ref. 7, 8) and therefore new biomarkers are needed that may serve as diagnostic/prognostic/predictive biomarkers or targets of therapy” is commonly seen and widely accepted, yet it may not be the best way to attract your readers and to motivate them to keep on reading.

Every original research paper does not only provide novel data, which is of course its main task, and this is why these papers are ranked highly in the scientific world, but it also has the task to educate, summarize and clarify concisely what has been known before. After reading your paper, the reader should be well acquainted with the data on the subject that your study builds on and of course, what news have been discovered and what has been added to the stock of the known by your research. The distribution of this information of prior works on the subject is to be carefully balanced between the introduction and the discussion. It is unwise to repeat these parts unnecessarily—this is boring and only increases the word‐count. The introduction should not give away results but explain the principal research questions and serve as a teaser to read on. Usually, 1–1.5 pages are sufficient to achieve this.

### 2.3 The Material and Methods Section

Here you describe the materials and methods used in your study, which is usually relatively easy to accomplish. Use a clear structure with subheadings, if appropriate and be as accurate as possible. In the following I will not list all pitfalls of all possible methods. Suffice it to say, that every method you use should be described in a way that the reader should be able to reproduce your findings. Science that cannot be verified by others is not worth publishing! Citing a method and adding “with minor modifications” is not good enough unless you describe the “minor modifications” you applied in sufficient detail. It is beyond the scope of this little paper to explain the pitfalls of every possible method, so I will restrict it to methods that are very commonly found in pathology papers.

#### 2.3.1 Demographics

The demographics of your patients should be precise, for example, in a Table 1. Experienced editors will be able to estimate the quality of your paper but having a look at “Table 1” – so beware of “reviewer 3” and be accurate and exhaustive. Was there any selection bias in your cohort? Either upfront or after your experiments? It is not uncommon to see unplausible descriptions of cohorts that must have undergone a selection, for exampe, if a large university center analyzes muscle invasive bladder cancer from a 10 year period and comes up with only 70 cases in total. The reader cannot know if the underlying selection could potentially bias the results (if e.g., only very large tumors with ample material were used for research), or if the selection is not associated with clinico‐pathological parameters, because for example, only the first 7 cases from each year were selected to cover a larger time period.

#### 2.3.2 Ethics Statement

Mentioning and specifying an ethics approval of your study from your local ethics committee is increasingly demanded by many journals and should be stated here. It would deserve an open discussion, what kind of ethics approval is appropriate for retrospective studies that are so common in pathology departments, as I see the increasing demand for prospective bio‐marker specific patient consents is suffocating pathology research. As I see it, demanding prospective consents in general is not a particularly high standard of ethics, but the end of retrospective research—what I consider as truly unethical—*cui bono*?

#### 2.3.3 Tissue Microarrays

Tissue microarrays are commonly used in descriptive analyses of pathologists for convenient high‐throughput profiling of cohorts. Often this is described very briefly and inadequately by citing a previous paper (“A TMA of the tumor cohort was used as described (NN)”). If you take the trouble to follow this up, you may find that even in the very first publication of the TMA in question, relevant information is not disclosed. Who selected the tumor areas of the donor blocks? Was the tumor sampled in the center or the periphery or both? What was the core size? How many cores were retrieved? Did the number of cores differ from case to case? If so, what is the number of mean/median cores? How many recipient blocks were needed in total? Did you include any control tissues? When was the TMA constructed, how was it stored? Was it freshly sectioned or did you use older sections? Were they dipped in paraffin? How were these stored (e.g., at room or fridge temperature)?

#### 2.3.4 Immunohistochemistry

Many pathology papers use some kind of Immunohistochemistry. Describe the technique in detail: pretreatment? Which kind of buffer? Citrate or Tris buffered saline? Something else? Pressure cooking? Microwave? Times? Automated platform? Name of protocol? Antibodies should be described with the clone name (if monoclonal), commercial supplier (including catalog number), and titration used. It is not so rare to see ambiguous descriptions like “the monoclonal NNN Antibody was obtained from mmm”, only to find that mmm has a dozen monoclonal antibodies in their portfolio. Specify the diluent, too. Has the specificity of the used antibody clone been demonstrated before? If not, it may be a good idea to do so and to include knockdown experiments with western blots with this antibody. Also, preincubation with the immunogenic peptide/protein in excess (e.g., 10:1) are widely accepted to rule out off‐target binding of an antibody: if the antibody is fully blocked, it should not display any relevant signal on tissues. Have you used (positive/negative) controls with your immunostainings?

##### 2.3.4.1 Evaluation of Immunohistochemistry

Describe how you evaluated and categorized your immunostainings. Which cell type was looked at? What subcellular compartment? The system of evaluation depends on the heterogeneity and intensity of marker immunoreactivity: few markers are either negative or positive, hence some system of semi quantitation is needed. In homogenously expressed markers, a simple system, categorizing staining intensity into negative, weakly, moderately or strongly positive (0–3+) may be fine. More heterogenous markers demand a system that incorporate the area of positivity and the staining intensity. Typically, area and intensity are categorized (e.g., area: 0 vs. 1 = 1%–9% vs. 2 = 10–50 vs. 3 = 51–80, and 4 = 81%–100%; intensity 0–3) and the product is calculated (range 0–12 in this case) [5]. The Allred score pursues a similar approach by adding intensity and area, with different cut‐off values though (range 0–8) [6]. The most meticulous approach is to estimate the percentage of weakly, moderately and strongly stained cells, which may be summed up in a weighted manner to obtain the so called HSCORE (=%weak immunoreactivity +2* %moderate immunoreactivity+3*% strong immunoreactivity). The system chosen is critically dependent on the biology of the marker under consideration. It does not make sense, to evaluate the staining intensity alone of a proliferation marker like Ki‐67 or to evaluate unplausible subcellular compartments (e.g., cytoplasmic staining of Ki‐67 or nuclear HER2, etc.). Did several investigators read the slides? How did you deal with discrepancies?

#### 2.3.5 Statistics

Briefly describe, which statistical tests you applied in your study and which software package was used. Define statistical significance (most commonly *p* < 0.05). It is often helpful and enriching to involve statisticians early in the project phase, so consider contacting your statistics department, because there is more to statistical life than crosstables and Kaplan–Meier curves.

## 3 Results

This part is usually easy to construct, hence not few authors start with it when writing a paper. Present the data in a structured manner, make use of numbers, headers and paragraphs for clarity. Some authors want to take the reader by the hand and explain in detail the idea why this or that section of data was generated (“We then wanted to see, if this protein is expressed also in other tumors and stained…”, “with the statistical evaluation we wanted to clarify, if there were any links between…”, etc.). It may be a matter of taste, but my recommendation is to omit all this type of superfluous procedural information and let the data speak for itself. Be brief and precise. Also, avoid all comments and interpretations, this belongs to the discussion exclusively.

Tables are a convenient way to present data, typically one table will present clinicopathological parameters, another the distribution of biomarker data in association with the former (this may include first statistical tests), further tables may present additional data like statistical tests, multivariable test, etc. Avoid redundancies of text and tables. If you describe the clinic‐pathological parameters in the text, do not care for a table with identical data. Tables are not included in the results text, but put to the appendices of the paper following the references. Put one table per page for greater clarity and provide a headline for every table (e.g., “table 1. Clinicopathological parameters of the tumor cohort”).

Illustrations should be appealing, informative and of high quality, especially if histology is depicted. For histology images, I found photographs taken at 200× best suited for publication, this still shows the tissue context and already allows to appreciate cytological details. Ideally, take all images at the same magnification. It is not necessary to take low power pictures from TMA cores to illustrate the TMA technique, since this technique has been around for > 20 years [7].

## 4 Discussion

The discussion part takes most people longest to write and is considered the most difficult but also the most creative section of the paper. In the discussion, a brief reiteration of the results is provided, but this time in a commented fashion, including existing data of other studies, even highly speculative hypotheses may be formulated in this part, leaving you a lot of freedom—which is also a bit scary to some. No reason for despair, but appropriate respect: the discussion is that magical part of the paper, where scientific data becomes knowledge by sorting and weighing it and where hypotheses, theories, or results are finally formulated. The discussion is also the part, where you bring your data to the market and present it in polished form, so care thoughtful for good readability and tell the story well. Does it have a good flow, or are some passages clumsy and less well integrated? Even marketing language is permissible, if not overused. “We carefully established a validated immunohistochemistry assay for the detection of xyz” does sound more elaborated that “we stained xyz,” does it not?

In citing other studies refer to the original papers, whenever possible. Citing review papers for general aspects can be quite a life saver, so do not refrain from using them occasionally, but do not cite the very same review paper too repetitively in your discussion. Sometimes, editors demand to replace older citations for newer ones to make the paper appear more contemporary. I personally consider this unscientific, as the credit for describing something first is thus withdrawn from these authors. So, maintain fairness and cite authors in correct historical order, even if the first description of something dates back a while.

Before writing the discussion, it is helpful to sort your thoughts and to recapitulate your findings. Help yourself, sharpen your pencil and jot down some bullet points. What was known before? What did your study add? Compile a list of thoughts you want to express or work on in your discussion. Take your time meditating on this.

A typical starting point could be to summarize what was done in this study and to briefly explain, which scientific or medical need this addresses. What has prompted you to conduct this study? Take care not to simply repeat from your introduction, or at least not too much!

**Questions to Structure Your Discussion**

- What are the *main findings*? Did they match your expectations?
- *Compare your data to previous studies* on this topic. What are similarities, what are differences?
- *Speculate on explanations for differences* (differences in cohorts? Technique? Evaluation scheme?).
- *What is known about your findings in other tumors*? Is there a recognizable pattern, for example, if a protein is associated with a histological or biological subtype? Markers found in one type of squamous cell carcinoma may also play a role in squamous cell carcinomas of other organs, etc.

**Further Aspects**

- Is the tumor type under scrutiny steroid hormone driven, your marker may also play a role in other steroid hormone dependent tumors. Are there any papers indicating a link?
- If your data is difficult to interpret, think harder what to make of this, before you just surrender to describe it merely as “complex” or “challenging.”
- Shed light on your methods, too. Is, for example, the immunohistochemistry easy to read and the groups of low vs. high expression clearly separable or are they quite close together, which might make it difficult to reproduce your findings?
- If your data suffers from a high dropout rate, mention this and discuss possible reasons.

A typical section for later paragraphs in the discussion—and one that some journals even explicitly require—is to list the strengths and weaknesses of the study. This actually provides a valuable opportunity to become aware of these aspects.

**Common Weaknesses in Pathology Studies**

- *Monocentric cohorts*: Data often comes from a single center, limiting generalizability.
- *Selection bias*: cases are compiled over a longer period of time and are not consecutively collected.
- *Low case numbers*: A small number of cases can reduce the study's significance and statistical power.
- *Retrospective study design*: Retrospective studies are more prone to biases and limitations in data scope.
- *Insufficient follow*‐*up data*: A short or incomplete follow‐up period can complicate the interpretation of results.
- *Weak endpoints*: Outcomes like recurrence‐free or progression‐free survival are often less meaningful than overall survival or disease‐specific survival—after all, death is quite a definitive endpoint, isn't it?
- *Lack of assay validation*: Without validation, reproducibility remains questionable.
- *Missing second biological level*: Observations on the proteomic level, for example, remain incomplete without complementary data on the genomic or transcriptomic level.

**Common Strengths**

- *Highly specific tumor cohort*: A well‐defined cohort, such as one derived from a clinical study with very careful surveillance, increases the study's relevance.
- *Large case numbers*: A high number of cases enhances statistical reliability.
- *Professional statistical analysis*: Robust statistical analysis strengthens the credibility and validity of the findings (hence the recommendation to involve statisticians).
- *Originality of the research question*: A novel and relevant research question makes the study stand out.

Reflecting on these points allows you to create a balanced discussion and position your study convincingly. Including such a section not only highlights your critical engagement with your own work but also demonstrates scientific integrity.

The final paragraph of a discussion is typically the *conclusion*, where you succinctly encapsulate the essence of your study. This section serves as a concise summary of your main findings, highlighting the key contributions your research has made to the scientific field. Additionally, it is an opportunity to point out any limitations and to outline the questions that remain unanswered, thus paving the way for future research. This paragraph should be impactful yet brief, leaving the reader with a clear understanding of the significance of your work and its potential implications.

## 5 Acknowledgments

Here you ought to mention all helping hands that do not fulfill the formal criteria of authorship (e.g., “We thank nnn for excellent technical support.”). Funding sources should be referenced here as well. If necessary, you can also thank your parents, partners or friends for their endless support and understanding.

## 6 Reference

If you used a bibliography program, it should be easy to list all references in the required order and style of your target journal. Otherwise, you would need to do this manually, which is a nightmare.

## 7 Figure Legends

Describe in short statements, what can be seen on your illustrations, for example, tissue type and staining. Use arrows or asterisks in images to hint at details but do this sparingly. Does the image have a bar to illustrate lengths? Alternatively, the magnification should be mentioned.

## 8 Final Phase—Submitting

You have compiled everything, the first draft of the paper is finished, all coauthors are informed and have corrected your paper and you think about a promising target journal. This should actually be done rather sooner than later, as the specifics of the journal will impact the details of your paper draft (word count, structure, etc.). Many factors influence your choice of the journal [8]. Generally speaking, prestigious journals with high impact factors ensure the greatest visibility, but are very selective in accepting papers, as they can choose from the worlds very best scientists—so having a paper in Nature or Science is never possible by pure chance… Pathologists often publish in pathology or oncology journals, which are lower in their impact factor, but generally easier to attain. As most papers nowadays will be found in Pubmed/Medline, the impact factor, even though it may still be important for evaluations of scientific productivity in university rankings, has actually become less important. Only very few scientists regularly read a couple of major journals consecutively to remain up to date. The more contemporary way is to deposit a couple of keywords within Pubmed or Google Scholar, so you get alerted automatically, whenever a paper on, for example, your favorite gene, tumor or author has come out—irrespective of the particular journal. Again, take this as a reminder to the importance of the choice of suitable key words. If your findings are actually more relevant for clinicians, it may be a good idea to pick a clinical journal for a start. If the results are more practice changing in diagnostic histopathology, aim for a pathology journal. If your data is more experimental, a wide choice of scientific journals may be open for your work.

Most journals require a letter to the editor to accompany your submission. Again, this is a great chance to attain the attention and the support of the editor. Editors browse through dozens of papers a week, so standing out in a positive sense should be your aim. Similar to the abstract, you should explain the open question/medical need and summarize the main findings. You may also give further information about the history of this paper, explain what inspired you to do this research and why this paper will be of interest to the readership of the target journal. Naming potential reviewers is increasingly required in many journals, you may also specifically exclude colleagues as reviewers, but beware, the editor may become particularly curious to learn, what your fiercest enemy thinks about your work, so this option is a double‐bladed sword.

Good luck with your research and have fun writing papers!

## Conflicts of Interest

The author declares no conflicts of interest.

## Data Availability

Data sharing is not applicable to this article as no new data were created or analyzed in this study.

## References

[1] S. P. Turbek , T. M. Chock , and K. Donahue , “Scientific Writing Made Easy: A Step‐By‐Step Guide to Undergraduate Writing in the Biological Sciences,” Bulletin of the Ecological Society of America 97 (2016): 417–426.

[2] B. J. Hoogenboom and R. C. Manske , “How to Write a Scientific Article,” International Journal of Sports Physical 7, no. 5 (2012): 512. PMC3474301 23091783

[3] F. Ecarnot , M. F. Seronde , R. Chopard , and F. Schiele , “Writing a Scientific Article: A Step‐By‐Step Guide for Beginners,” European Geriatric Medicine 6 (2015): 573–579.

[4] A. Cunha , B. dos Santos , Á. M. Dias , A. M. Carmagnani , B. Lafer , and G. F. Busatto , “Success in Publication by Graduate Students in Psychiatry in Brazil: An Empirical Evaluation of the Relative Influence of English Proficiency and Advisor Expertise,” BMC Medical Education 14 (2014): 238. 25373331 10.1186/1472-6920-14-238 PMC4289391

[5] W. Remmele and H. E. Stegner , “Recommendation for Uniform Definition of an Immunoreactive Score (IRS) for Immunohistochemical Estrogen Receptor Detection (ER‐ICA) in Breast Cancer Tissue,” Pathologe 8 (1987): 138–140. 3303008

[6] D. C. Allred , M. A. Bustamante , C. O. Daniel , H. V. Gaskill , and A. B. Cruz , “Immunocytochemical Analysis of Estrogen Receptors in Human Breast Carcinomas. Evaluation of 130 Cases and Review of the Literature Regarding Concordance With Biochemical Assay and Clinical Relevance,” Archives of Surgery 125 (1990): 107–113. 1688490 10.1001/archsurg.1990.01410130113018

[7] J. Kononen , L. Bubendorf , A. Kallioniemi , et al., “Tissue Microarrays for High‐Throughput Molecular Profiling of Tumor Specimens,” Nature Medicine 4 (1998): 844–847. 10.1038/nm0798-844 9662379

[8] L. A. Maggio , N. Chtena , J. P. Alperin , L. Moorhead , and J. M. Willinsky , “The Best Home for This Paper: A Qualitative Study of How Authors Select Where to Submit Manuscripts,” Perspectives on Medical Education 13 (2024): 442–451. 39290445 10.5334/pme.1517 PMC11405847

